# A real-time 3D electromagnetic navigation system for percutaneous transforaminal endoscopic discectomy in patients with lumbar disc herniation: a retrospective study

**DOI:** 10.1186/s12891-022-05012-6

**Published:** 2022-01-17

**Authors:** Boyu Wu, Tanjun Wei, Zhipeng Yao, Sai Yang, Yawei Yao, Chengwei Fu, Feng Xu, Chengjie Xiong

**Affiliations:** 1 Orthopaedic Department, General Hospital of Central Theater Command of PLA, Wuhan, 430070 China; 2 Hunan University of Chinese Medicine, Changsha, 410208 China; 3 Southern Medical University, Guangzhou, 51000 China; 4 Wuhan University of Science and Technology, Wuhan, 430065 China; 5 Guangzhou University of Chinese Medicine, Guangzhou, China

**Keywords:** Electromagnetic navigation, Percutaneous transforaminal endoscopic discectomy, Lumbar disc herniation, Learning curve, Radiation exposure

## Abstract

**Background:**

In this study, we present a novel electromagnetic navigation (EMN) system for percutaneous transforaminal endoscopic discectomy (PTED) procedure. The objective of this study was to investigate the safety and effectiveness of the PTED with the assistance of the EMN system and compare it with the conventional PTED with the assistance of fluoroscopic guidance (C-arm).

**Methods:**

The clinical data of 79 patients (32 in EMN group and 47 in C-arm group) undergoing PTED for lumbar disc herniation (LDH) from January to September of 2019 were analyzed retrospectively. The radiation time, puncture time, operation time, visual analog scale (VAS), Oswestry disability index (ODI), modified MacNab criteria, and radiological parameters were recorded in both groups.

**Results:**

Radiation time, puncture time, and operation time were significantly reduced in the EMN group compared with the C-arm group (*P* < 0.05). Compared with the C-arm group, a steeper learning curve was observed in the EMN group. There were no significant differences between the two groups regarding VAS and ODI scores at different time points (*P* > 0.05). The satisfaction rates of the EMN and C-arm groups were 90.63 and 87.23%, respectively, but no significant difference was found between the two groups (*P* > 0.05). There was no significant difference regarding translation and angular motion between the two groups at preoperation and postoperation (*P* > 0.05).

**Conclusions:**

The EMN system can be applied to facilitate the PETD procedure. It can significantly reduce the intraoperative radiation time, puncture time, and operation time, and reshape the learning curve of PTED. Due to limitations of a retrospective study, results may need validation with larger prospective randomized clinical trials.

## Background

Minimally invasive spine surgery (MISS) has been gained popularity in recent years. It is widely accepted as a safe and effective technique [1–3]. It can limit soft tissue retraction and dissection, which results in decreased postoperative pain and improved functional recovery [4, 5]. With the advancement of MISS, percutaneous transforaminal endoscopic discectomy (PTED) has become an alternative approach for treating lumbar disc herniation (LDH) [6, 7].

Nevertheless, conventional PTED is considered as a technical demanding procedure with a long learning curve. It is time-consuming and experience-dependent [8, 9]. Repeated fluoroscopy is required to perform the puncture in a trial-and-error approach. It would not only increase operation time, but also increase the risk of nerve root injury and radiation exposure to medical staff and patients [10, 11]. Thus, shortening the learning curve and reducing radiation exposure are the common goal for surgeons. Recently, several attempts have been developed to achieve this goal, including ultrasound volume navigation, optical navigation, electromagnetic navigation (EMN) etc. [12–14].

With the aid of the EMN system, surgeons can accurately and safely track the position of surgical instruments based on preoperative computed tomography (CT) images and intraoperative radiographs [15–17]. EMN-assisted PTED has been reported to treat patients with LDH in a series of cases with favorable clinical outcomes [14]. However, this study was not a case-control study, and it is difficult for us to evaluate the efficiency and safety of EMN-assisted PTED compared with conventional PTED (C-arm assistance). Therefore, a retrospective study was conducted to compare the efficiency and safety between EMN-assisted PTED and C-arm assisted PTED.

## Methods

### Data collection

This retrospective study was performed in accordance with the Helsinki Declaration and has been approved by the Ethics Committee of the General Hospital of Central Theater Command of PLA (Ethics No. [2021] 037–01). Written informed consents was obtained from each patient. We retrospectively collected the clinical data of 79 patients with LDH who underwent PTED between January 2019 and September 2019 at our hospital. Patients were divided into two groups according to the different surgical methods: EMN group (32 patients underwent PTED assisted with EMN guidance) and C-arm group (47 patients underwent PTED guided by fluoroscopy). In our department, we provided patients with all surgical details of both surgery, including the surgical procedures, total cost and possible complications, and the final selections were made by the patients. All surgeries were performed by two surgeons with a similar medical background.

The inclusion criteria for the study were as follows: 1) age between 18 and 65 years; 2) single LDH (limited to L4-5 or L5-S1); 3) the type of disc herniation was central or para-central; 4) patients had symptoms of LDH, such as low back pain, lower extremity pain or numbness; 5) conservative treatments failed within 3 months. The exclusion criteria were as follows: 1) lumbar instability was observed on flexion-extension radiographs; 2) combined with central canal stenosis, calcified herniated disc, cauda equina syndrome, lumbar fracture, or active spinal infection; 3) previous lumbar surgery history; 4) patients who can’t tolerate surgery.

### Surgical procedure

#### EMN group

**Fig. 1:**
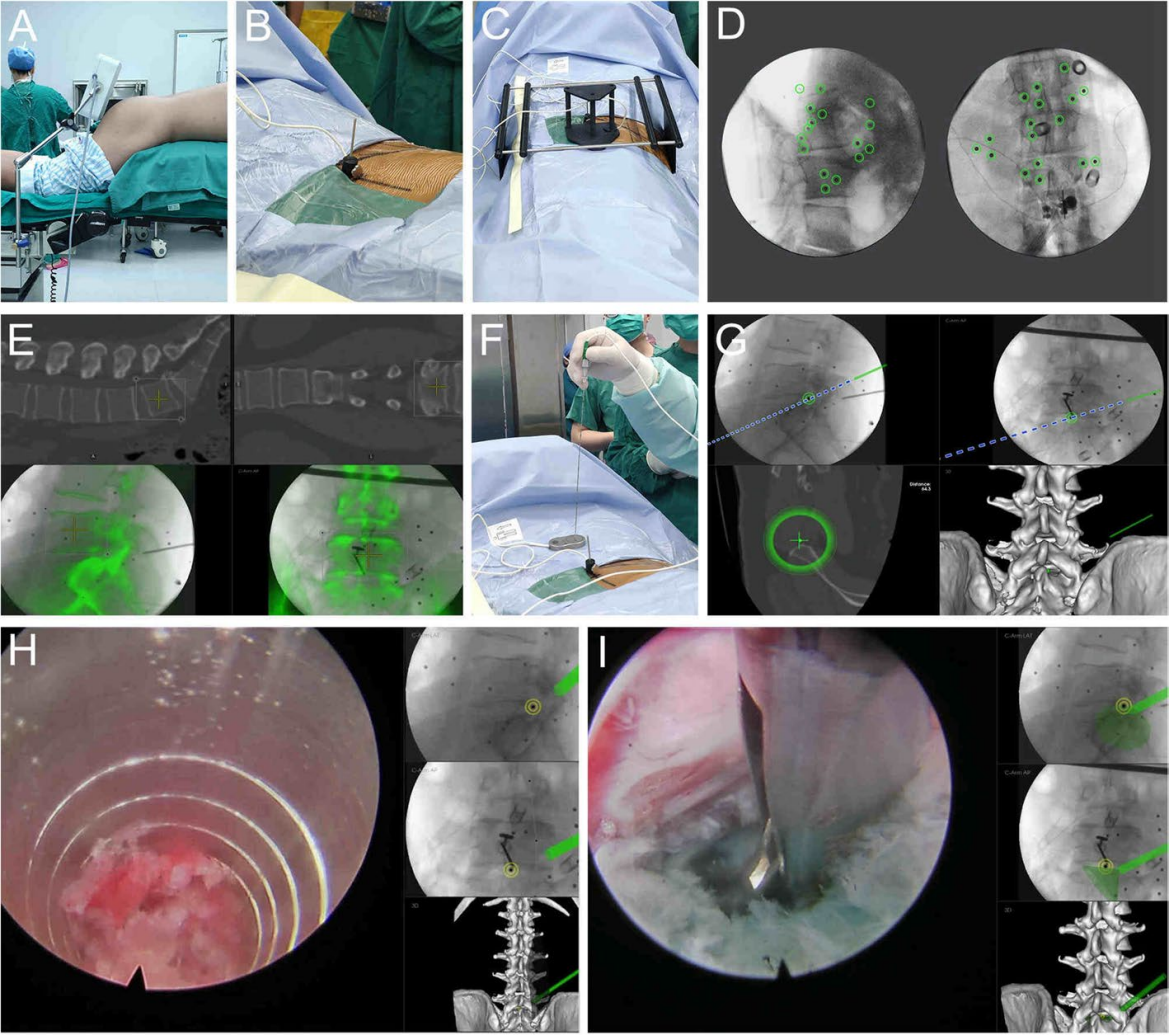
The procedure of PTED assisted by the EMN system. **a** The electromagnetic field generator was placed in the caudal site of the surgical area. **b** The patient tracker was fixed on the adjacent spinous process of the operative level. **c** The mapper bridge was placed in the surgical area. **d** The fluoroscopy images were transmitted to the EMN system for automatic registration. **e** The intraoperative two-dimensional fluoroscopy images were used to match the preoperative three-dimensional CT images. **f** Surgical instruments were registered. **g** The percutaneous puncture was performed under EMN guidance. **h** The foraminoplasty was done under the surveillance of navigation and endoscope. **i** Discectomy was performed under EMN guidance. PTED, percutaneous transforaminal endoscopic discectomy; EMN, electromagnetic navigation

All procedures are performed with the TESSYS Isee full-endoscopic system (Joimax, Karlsruhe, Germany) under the guidance of the EMN system (Fiagon, Berlin, Germany). The patients were placed in a prone position on a radiolucent operating table. The electromagnetic field generator was placed on the caudal side of the patient (Fig. 1a). After local anesthesia, a K-wire was anchored on the adjacent spinous process of the index level. Then the K-wire attached with the patient tracker was connected to the EMN system (Fig. 1b). The mapper bridge was placed over the surgical area (Fig. 1c). The fluoroscopic images were obtained and transmitted to the EMN system for automatic registration (Fig. 1d). After confirming the registration, intraoperative two-dimensional (2D) fluoroscopic images were adjusted to match the preoperative 3D CT images (Fig. 1e). An intraoperative real-time 3D navigation image was reconstructed. The registration of endoscopic surgical instruments was performed before the endoscopic manipulation (Fig. 1f). In 3D navigation mode, the sensor wire could be mounted into different surgical instruments (spinal needle, guiding rod, reamer, and endoscope).

The optimal entry point and puncture pathway can be determined with the assistance of the EMN system. Following local anesthesia, an 18G needle was inserted according to the designed pathway (Fig. 1g). The guidewire was introduced, the needle was removed, and a small incision was made around the guidewire. The sequential dilators were inserted over the guidewire. After graded dilation, the guiding rod was introduced along the guidewire into the target site. A half-serrated working cannula was passed through the guiding rod, which was then removed. An endoscope with irrigation system was inserted along the half-serrated working cannula, and foraminoplasty was performed by using a reamer under endoscopic surveillance (Fig. 1h). Following foraminoplasty, the half-serrated working cannula was replaced with a standard transforaminal working cannula. The remaining endoscopic procedures were performed as the routine protocol (Fig. 1i). Under the EMN guidance, the depth and location of the endoscope and relevant anatomical structures could be precisely presented on the monitor during surgery.

#### C-arm group

All procedures are performed with the TESSYS full-endoscopic system (joimax, Karlsruhe, Germany) under fluoroscopic guidance, as described in the previous study [18].

### Clinical assessment

Baseline characteristics, including sex, age, body mass index (BMI), operative level, and follow-up duration were collected. Intraoperative data, including the radiation time, puncture time, and operation time were recorded. “Puncture time” was defined as the duration between the initial needle puncture and the establishment of working cannula. The visual analogue scale (VAS) and Oswestry disability index (ODI) scores were used to assess clinical outcomes. The overall satisfaction was assessed using modified MacNab criteria. Follow-up data were collected at 1-, 3-, 6-, and 12-month postoperatively.

**Fig. 2:**
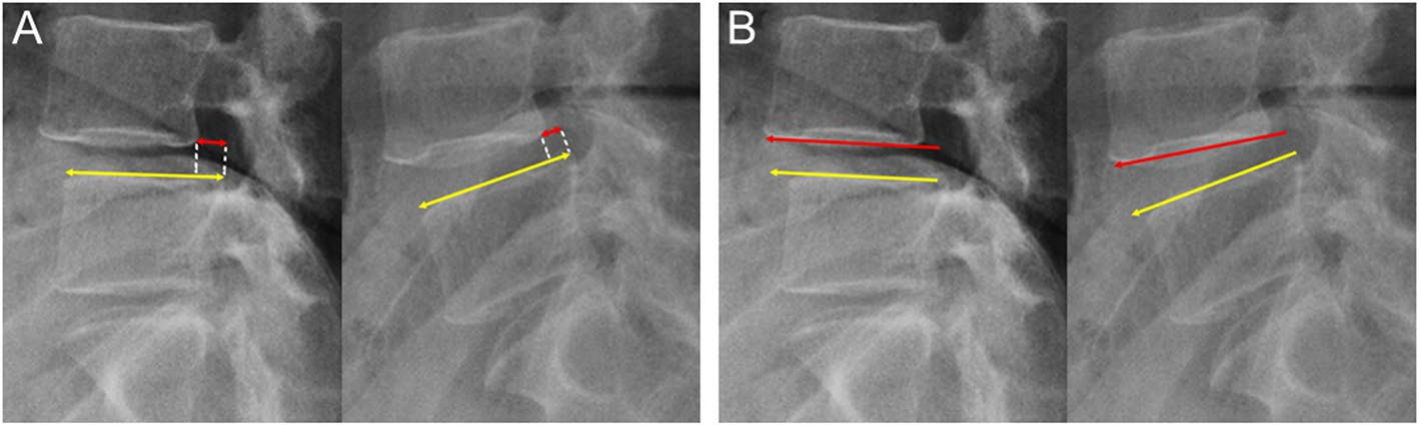
Measurement of radiological parameters. **a** Angular motion: the change in intervertebral angle in flexion/extension. **b** Translation: the change in slip distance in flexion/extension

All patients underwent lumbar flexion-extension radiographs at preoperation and the latest follow-up. To determine the lumbar stability, we measured and assessed the translation and angular motion using flexion-extension radiographs (Fig. 2). According to the literature, dynamic instability was defined as translation motion more than 3 mm and angular motion (between adjacent vertebral endplates) greater than 10° [19, 20].

### Statistical analysis

Data were analyzed with SPSS 24.0 (SPSS, Chicago, IL). Continuous variables were expressed as means±standard deviation. Student’s t-tests was used to compare the averages of continuous variables, such as age, BMI, follow-up duration, radiation time, puncture time, operation time, VAS, ODI scores, translation, and angular motion. Chi-square tests were applied to compare the categorical variables, such as sex, operative level, satisfaction rate, and complication rate. *P*-value < 0.05 were considered to be significant.

## Results

### Preoperative baseline characteristics

A total of 79 patients (32 in the EMN group and 47 in the C-arm group) were included in this study. There were no differences regarding baseline characteristics between the two groups (*P* > 0.05). (Table 1).

**Table 1:** Comparison of baseline characteristics between the two groups

Characteristics	EMN (*n* = 32)	C-arm (*n* = 47)	*P* value
Age (years)	46.88 ± 11.10	43.13 ± 10.30	0.13
Gender (male/female)	18/14	25/22	0.79
BMI (kg/m^2^)	22.82 ± 3.74	23.69 ± 3.70	0.31
Operative level (L4-L5/L5-S1)	19/13	30/17	0.69
Follow-up (months)	13.66 ± 1.07	14.04 ± 1.20	0.15

*BMI* body mass index

### Clinical outcomes

The radiation time (4.50 ± 0.99 s), puncture time (26.13 ± 14.29 min), and operation time (72.97 ± 25.24 min) in the EMN group were significantly shorter than those (28.61 ± 12.19 s; 44.15 ± 13.47 min; 94.21 ± 20.93 min) in the C-arm group (*P* < 0.05) (Table 2).

**Table 2:** Comparison of surgery-related records between the two groups

Characteristics	EMN (*n* = 32)	C-arm (*n* = 47)	*P* value
Radiation time (s)	4.50 ± 0.99	28.61 ± 12.19	< 0.001
Puncture time (min)	26.13 ± 14.29	44.15 ± 13.47	< 0.001
Operative time (min)	72.97 ± 25.24	94.21 ± 20.93	< 0.001

**Fig. 3:**
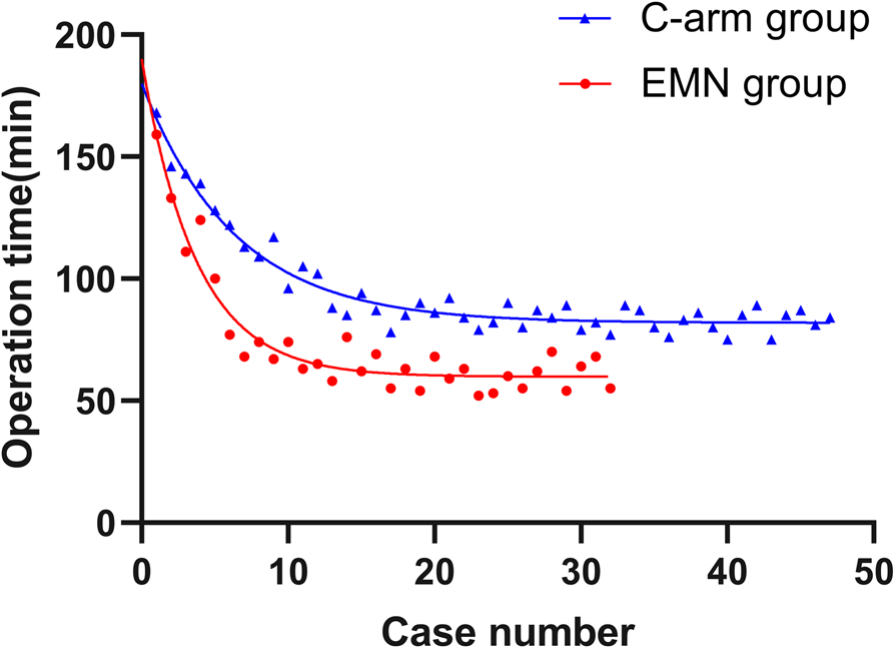
The learning curves for PTED via the EMN and C-arm approaches. PTED, percutaneous transforaminal endoscopic discectomy; EMN, electromagnetic navigation

The model of the learning curve was established according to operation time. The learning curve was steeper in the EMN group compared with the C-arm group. (Fig. 3).

**Fig. 4:**
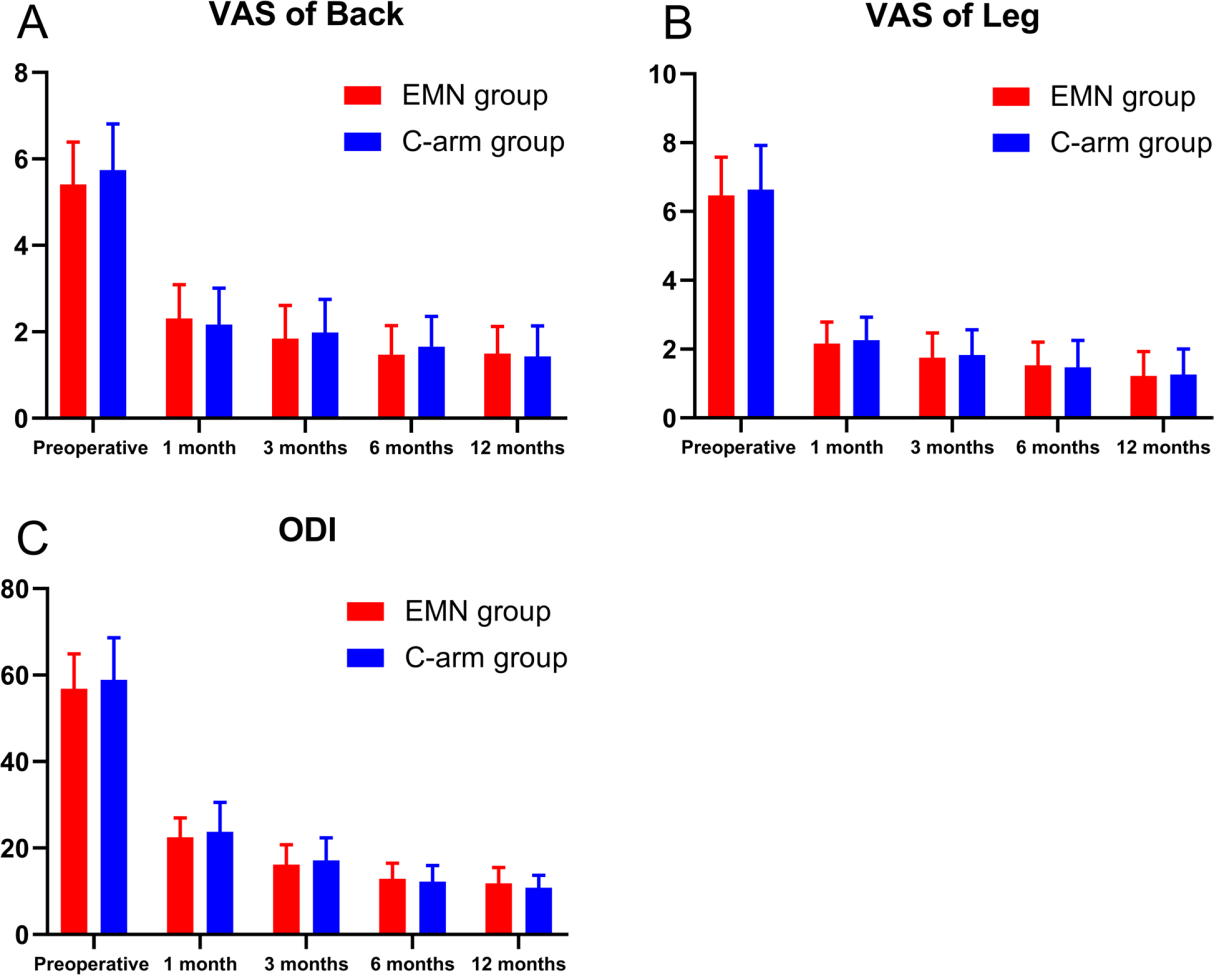
Comparison of VAS score of back (**a**), VAS score of leg (**b**) and ODI (**c**) at different time points. VAS, visual analogue scale; ODI, Oswestry Disability Index

The VAS and ODI scores following surgery improved in both the EMN group and C-arm group (*P* < 0.05). No significant differences were found regarding VAS and ODI scores between the two groups at different time points (*P* > 0.05). (Table 3 and Fig. 4).

**Table 3:** Comparison of VAS and ODI scores between the two groups

Characteristics	EMN (*n* = 32)	C-arm (*n* = 47)	*P* value
VAS of Back			
Preoperative	5.41 ± 0.98	5.74 ± 1.07	0.16
1 month	2.31 ± 0.78	2.17 ± 0.84	0.45
3 months	1.84 ± 0.77	1.98 ± 0.77	0.44
6 months	1.47 ± 0.67	1.66 ± 0.70	0.23
12 months	1.50 ± 0.62	1.43 ± 0.71	0.63
VAS of Leg			
Preoperative	6.47 ± 1.11	6.64 ± 1.28	0.54
1 month	2.16 ± 0.63	2.26 ± 0.67	0.51
3 months	1.75 ± 0.72	1.83 ± 0.73	0.63
6 months	1.53 ± 0.67	1.47 ± 0.78	0.71
12 months	1.22 ± 0.71	1.26 ± 0.74	0.83
ODI			
Preoperative	56.81 ± 8.11	58.89 ± 9.73	0.32
1 month	22.50 ± 4.49	23.74 ± 6.81	0.37
3 months	16.19 ± 4.55	17.15 ± 5.24	0.40
6 months	12.89 ± 3.63	12.21 ± 3.76	0.44
12 months	11.81 ± 3.68	10.81 ± 2.88	0.18

*VAS* visual analogue scale, *ODI* Oswestry Disability Index

According to the modified MacNab criteria, the satisfaction rates were 90.63% in the EMN group and 87.23% in the C-arm group; no significant difference was found between the two groups (*P* > 0.05). (Table 4).

**Table 4:** Comparison of MacNab evaluation between the two groups (n, %)

Groups	n	Excellent	Good	Fair	Poor
EMN	32	20 (62.50)	9 (28.13)	2 (6.25)	1 (3.13)
C-arm	47	26 (55.32)	15 (31.91)	4 (8.51)	2 (4.26)
*P* value	0.93				

### Radiologic findings

No patients exhibited lumbar instability, as determined by flexion-extension radiographs. There was no significant difference in the translation and angular motion between the two groups at the preoperation and postoperation (*P* > 0.05). The postoperative translation and angular motion increased in two groups compared with those at preoperation, but the difference was not statistically significant (*P* > 0.05). (Table 5).

**Table 5:** Comparison of radiological parameters between the two groups

Characteristics	EMN (*n* = 32)	C-arm (*n* = 47)	*P* value
Translation			
Preoperative (mm)	1.42 ± 0.60	1.35 ± 0.57	0.59
Postoperative (mm)	1.65 ± 0.62	1.49 ± 0.63	0.27
*P* value	0.16	0.24	
Angular motion			
Preoperative (°)	3.32 ± 1.68	3.58 ± 1.36	0.45
Postoperative (°)	3.87 ± 1.88	3.96 ± 1.92	0.85
*P* value	0.22	0.27	

### Operation complications and recurrence

There were no major complications (such as vascular injury, cerebral spinal fluid leakage, surgical infection, and lumbar instability) in 79 patients. Complications occurred in one case (3.13%) in the EMN group and three cases (6.38%) in the C-arm group, no significant difference was observed between the two groups (*P* > 0.05). One case in the EMN group and two cases in the C-arm group suffered from postoperative transient dysesthesia. Their symptoms were relieved following conservative management. One case in the C-arm group experienced symptomatic residual disc herniations, and this patient received a revision PTED surgery. There was one recurrent case in each group. Two of them underwent lumbar spinal fusion after a failed conservative management, and their symptoms were relieved at the latest follow-up.

## Discussion

PTED can offer many advantages compared to traditional open surgery, and it has been increasingly used for the treatment of LDH [4, 5, 21, 22]. In clinical practice, transforaminal needle placement was perceived to be the most critical and challenging step of this operation. It was predominantly dependent on repeated puncture and fluoroscopy in a trial-and-error way [13, 23]. Inappropriate punctures might increase the risk of nerve root injury and operation time [6, 13]. Therefore, there is a long learning curve to excel in PTED [8].

In recent years, some techniques have been developed to reduce the difficulty of this operation and shorten the learning curve. A self-made mechanical navigation tool has been applied to assist PETD, which decrease the x-ray radiation and reshape the learning curve of the operation [23, 24]. However, the accuracy of puncture can’t be guaranteed based on the evidence available. Later, a specially designed fluoroscope with an MRI-equipped operative suite was also applied to perform image-guided puncture. Whereas, this device was quite expensive. Moreover, the surgical manipulations were quite complicated and time-consuming [25]. Liu et al. [26] used the ultrasound volume navigation technique to guide posterolateral transforaminal puncture, and found a significant reduction in puncture time and intraoperative radiation exposure. However, additional training in ultrasound was needed before engaging in PTED. Optical navigation systems are widely used in MISS due to their highly accurate and well-established technology [13, 27]. However, a disadvantage of this system is the line-of-sight since the reflection spheres need to be observed by a camera, and the line-of-sight can be blocked by a solid barrier [28]. In addition, the reflection spheres are usually mounted in the handle of surgical instruments, so the flexible needle tips are difficult to track the target site reliably.

Therefore, the EMN system has been developed and applied in spine surgery due to the limitations of currently available techniques. The EMN system can integrate preoperative CT data with intraoperative fluoroscopic data, and reconstruct the intraoperative real-time 3D navigation image. Surgeons can obtain the detailed 3D anatomical structure of the spine and reduce the potential risk of neurological or vascular injury [13, 29]. The EMN system is based on the principle that a voltage is induced on the tip of the sensor, which is placed in an electromagnetic field [17]. It is composed of a field generator, patient tracker, mapper bridge, sensor wire, computer system, and matched surgical instruments [14, 29]. The magnitude of voltage depends on the spatial location and orientation of the sensor’s tip within the field [17]. The EMN system can provide higher spatial accuracy comparable with traditional optical navigation. The electromagnetic fields generated by the EMN system can penetrate the body and thus overcome the limitation of line-of-sight in the optical navigation system [28, 30]. Moreover, flexible surgical instruments such as spinal needles are compatible with this EMN system. It can accurately track the tip of various surgical instruments [31, 32].

So far as we know, the application of endoscopic spinal surgery assisted with the EMN system has only been reported in two studies [14, 29]. Similar to our study, it has been reported that the EMN system can significantly decrease radiation exposure. However, Lin et al. [14] described EMN-assisted PTED for patients with LDH in a case series, and it is not a well-designed case-control study. We can’t evaluate the efficiency and safety of EMN-assisted PTED compared with conventional PTED assisted with C-arm. Wu et al. [29] have introduced the application of the EMN system for percutaneous transforaminal endoscopic lumbar decompression in patients with lumbar spinal stenosis (LSS). However, the surgical technique and instruments applied for LSS are significantly different to those used in the PTED procedure for LDH. The application of the EMN system in this study might not guarantee its reliable application in the PTED procedure for LDH. Therefore, we conducted this study and compared the efficiency and safety between PTED assisted with the EMN system and C-arm in treating patients with LDH. The preliminary results demonstrated that the PTED assisted with EMN system can significantly reduce the intraoperative radiation, puncture, and operation time compared with conventional PTED assisted with C-arm.

Despite advantages of the PTED assisted with EMN system, there are still several limitations. Firstly, the ferromagnetic substances would cause electromagnetic field distortion, so only nonferromagnetic surgical instruments can be applied in the procedure [33]. Secondly, as a novel computer-assisted navigation system, the preoperative system set-up is time-consuming and needs the assistance of a professional technician. The system set-up time should be shortened with familiarity of this system and accumulation of cases. These results are consistent with the learning curve in the EMN group. Finally, the equipment of the EMN system is costly, which may increase the cost of surgery, thus hampering the generalization of this technique.

In this study, the EMN group has a steeper learning curve than the C-arm group. It was worth mentioning that the steeper learning curve was not always a negative outcome, since beginners can master key points of PTED quickly after a smaller number of cases [13, 23]. Our results show that the application of the EMN system could better cope with difficulty and promote mastery of PTED, and it also reshapes the learning curves of spinal endoscopic surgeons.

The clinical outcomes of our study are similar to those reported in the previous literature [13, 14, 23]. The satisfaction rates were 90.63 and 87.23% in the EMN and C-arm groups, respectively. There was a dramatic improvement in VAS and ODI scores in both groups after surgery, no differences were found between groups. It was reported that reduced operation time might result in better clinical outcomes [34]. However, there is no significant difference regarding neurological functional recovery in the long period. The earliest follow-up time point in this study was a 1-month time point. However, the 1-month time point is too long to evaluate the functional recovery, and the 1-week time point should be involved in future study.

There were several limitations in this study. First, this was a single-center retrospective study, which inevitably leads to selection bias. Second, the number of included cases was small, and the duration of follow-up time was short. Therefore, a prospective, multicenter prospective study with large samples should be awaited in the future. Moreover, there also could be potential bias due to the different surgical levels of two surgeons. Finally, in the EMN group, patients must undergo a preoperative 3D CT, which increased medical costs and radiation exposure to patients. However, it should be noted that the 3D CT was not necessary if the 2D navigation mode was selected in the procedure.

## Conclusions

The EMN system is a safe, effective and accurate tool that is compatible with PTED. It can significantly reduce intraoperative radiation time, puncture time, and operation time. Therefore, the EMN system reshapes the learning curves of PTED with a significantly lower learning difficulty. Due to limitations of a retrospective study, results may need validation with larger prospective randomized clinical trials .

## Data Availability

The datasets used and/or analysed during the current study available from the corresponding author on reasonable request.

## Abbreviations

MISS: Minimally invasive spine surgery
PTED: Percutaneous transforaminal endoscopic discectomy
LDH: Lumbar disk herniation
EMN: Electromagnetic navigation
CT: Computed tomography
BMI: Body mass index
VAS: Visual analogue scale
ODI: Oswestry disability index
LSS: Lumbar spinal stenosis
